# Monitoring of Regulatory T Cell Frequencies and Expression of CTLA-4 on T Cells, before and after DC Vaccination, Can Predict Survival in GBM Patients

**DOI:** 10.1371/journal.pone.0032614

**Published:** 2012-04-02

**Authors:** Brendan Fong, Richard Jin, Xiaoyan Wang, Michael Safaee, Dominique N. Lisiero, Isaac Yang, Gang Li, Linda M. Liau, Robert M. Prins

**Affiliations:** 1 Department of Neurosurgery, David Geffen School of Medicine at UCLA, University of California Los Angeles, Los Angeles, California, United States of America; 2 Department of Biostatistics, David Geffen School of Medicine at UCLA, University of California Los Angeles, Los Angeles, California, United States of America; 3 Jonsson Comprehensive Cancer Center, David Geffen School of Medicine at UCLA, University of California Los Angeles, Los Angeles, California, United States of America; 4 Brain Research Institute, David Geffen School of Medicine at UCLA, University of California Los Angeles, Los Angeles, California, United States of America; 5 Institute for Molecular Medicine, David Geffen School of Medicine at UCLA, University of California Los Angeles, Los Angeles, California, United States of America; University of Michigan School of Medicine, United States of America

## Abstract

**Purpose:**

Dendritic cell (DC) vaccines have recently emerged as an innovative therapeutic option for glioblastoma patients. To identify novel surrogates of anti-tumor immune responsiveness, we studied the dynamic expression of activation and inhibitory markers on peripheral blood lymphocyte (PBL) subsets in glioblastoma patients treated with DC vaccination at UCLA.

**Experimental Design:**

Pre-treatment and post-treatment PBL from 24 patients enrolled in two Phase I clinical trials of dendritic cell immunotherapy were stained and analyzed using flow cytometry. A univariate Cox proportional hazards model was utilized to investigate the association between continuous immune monitoring variables and survival. Finally, the immune monitoring variables were dichotomized and a recursive partitioning survival tree was built to obtain cut-off values predictive of survival.

**Results:**

The change in regulatory T cell (CD3^+^CD4^+^CD25^+^CD127^low^) frequency in PBL was significantly associated with survival (p = 0.0228; hazard ratio = 3.623) after DC vaccination. Furthermore, the dynamic expression of the negative co-stimulatory molecule, CTLA-4, was also significantly associated with survival on CD3^+^CD4^+^ T cells (p = 0.0191; hazard ratio = 2.840) and CD3^+^CD8^+^ T cells (p = 0.0273; hazard ratio = 2.690), while that of activation markers (CD25, CD69) was not. Finally, a recursive partitioning tree algorithm was utilized to dichotomize the post/pre fold change immune monitoring variables. The resultant cut-off values from these immune monitoring variables could effectively segregate these patients into groups with significantly different overall survival curves.

**Conclusions:**

Our results suggest that monitoring the change in regulatory T cell frequencies and dynamic expression of the negative co-stimulatory molecules on peripheral blood T cells, before and after DC vaccination, may predict survival. The cut-off point generated from these data can be utilized in future prospective immunotherapy trials to further evaluate its predictive validity.

## Introduction

Glioblastoma is one of the most lethal of human cancers, with very few long-term survivors and no definitive cures for this disease. These tumors invade and infiltrate the surrounding brain, making complete surgical excision impossible. They are also among the most radiation and chemotherapy resistant cancers, with a median survival of 12–18 months from initial diagnosis, even with surgery, radiation and chemotherapy [1], [2], [3], [4], [5]. The glioblastoma patient population has dismal outcomes and innovative approaches are desperately needed. Thus, glioblastoma remains a largely unmet medical need, and highlights the need for novel and effective therapies.

Recently, there has been a growing interest in applying tumor immunotherapy approaches to primary brain tumors, based on the recent FDA approvals for Sipuleucel-T in prostate cancer and Ipilumimab for metastatic melanoma [6], [7], [8], [9]. Immunotherapy is theoretically appealing because it offers the potential for a high degree of tumor-specificity, while sparing normal brain structures [10]. One such approach uses professional antigen-presenting cells, known as dendritic cells (DC), co-cultured with autologous tumor lysate or glioma-associated antigens to target these tumors immunologically. Initial studies of DC-based vaccine therapy for malignant gliomas have shown acceptable safety and toxicity profiles [11], [12], [13], [14], [15], [16], [17], [18], [19], [20], [21], [22], and multi-center randomized Phase II and III studies are currently underway.

While DC vaccine strategies have shown great promise [14], [15], [16], [21], [22], [23], there are still many barriers and uncertainties associated with this treatment modality. One of the prominent barriers of immunotherapy is the absence of biomarkers, imaging modalities and/or peripheral blood immune monitoring assays that can convey relevant information about anti-tumor immune responses elicited by the therapy. Many vaccine-based approaches consider the expansion of antigen-specific T cells, with functional activation characteristics, as the most important surrogates of efficacy. However, the majority of these immune monitoring strategies have not yielded an association with the clinical effects. The complexity of the treatments and patients, as well as the array of distinct monitoring assays, has not led to any uniform surrogate for immunotherapy.

Such history prompted us to analyze peripheral blood lymphocyte (PBL) populations for immunoregulatory factors that might be associated with predicting prognosis and monitoring patient progress after dendritic cell vaccination. We focused on the pattern of regulatory T (Treg) cell frequencies and negative co-stimulatory molecule expression on PBL, before and after DC vaccination. Treg cells play an essential role in lymphocyte development by maintaining tolerance and suppressing lymphocyte function [24]. Several groups have provided evidence that Treg cells accumulate in gliomas and suppress the anti tumor immune response [25], [26], [27], [28], [29], [30], [31], [32]. We also evaluated the dynamic expression of the negative co-stimulatory molecules (CTLA-4 and PD-1) on several cell populations. CTLA-4 and PD-1 both play essential roles in the regulation of peripheral tolerance by limiting T-cell activation and downstream signaling [33]. When CTLA-4 is upregulated on the surface of T-cells it can bind B7 co-stimulatory molecules with a higher affinity than CD28, preventing the initiation of T-cell activation [33]. In essence, CTLA-4 and Treg cells both function to down regulate the lymphocyte immune response. With a larger understanding of Treg cells and negative co-stimulatory molecules, we focused our research on examining their frequencies in peripheral lymphocyte populations. We found that *decreased* Treg cell populations and decreased expression of CTLA-4 on peripheral blood T cells, after DC vaccination, were correlated with longer survival in glioblastoma patients.

## Materials and Methods

### Patient Eligibility

This study focuses on 24 patients diagnosed with glioblastoma at our institution and treated with either autologous tumor lysate-pulsed (UCLA IRB #03-04-053, FDA IND #11053, clinical trial registration # NCT00068515) or glioma-associated antigen (GAA) peptide-pulsed (UCLA IRB #06-01-052, FDA IND #12966, clinical trial registration # NCT00612001) DC vaccination between 2003 and 2010. All patients provided written informed consent for studies approved by the UCLA Medical Institutional Review Board (IRB) prior to treatment. This informed consent was approved by the UCLA Medical IRB and given by patients for their experimental treatment, for a database that stored clinical data, and for research performed on remnant patient tissues. Patient inclusion/exclusion criteria have been published [14], [16], [34] and can be found at ClinicalTrials.gov for these studies (http://clinicaltrials.gov/).

### Preparation of Autologous Dendritic Cells and Pulsing with Glioma Antigen

Monocyte-derived DCs were established from adherent peripheral blood mononuclear cells (PBMC) obtained via leukapheresis, as we have recently published [16]. All *ex vivo* DC preparations were performed in the UCLA-Jonsson Cancer Center GMP facility under sterile and monitored conditions. Briefly, dendritic cells were prepared by culturing adherent PBMC in RPMI-1640 (Gibco) and supplemented with 10% autologous serum, 500 U/mL GM-CSF (Leukine®, Amgen, Thousand Oaks, CA) and 500 U/mL of IL-4 (CellGenix). For the GAA peptide trial, DC were additionally matured with a clinical-grade cocktail of 10 ng/ml TNF-α, 10 ng/ml IL-1β, 150 ng/ml IL-6 (all from CellGenix), and 1 μg/ml prostaglandin E_2_ (Sigma) for 24–48 hours [35]. Following culture, DCs were collected by vigorous rinsing and subsequently washed with sterile 0.9% NaCl solution. The purity and phenotype of each DC lot was also determined by flow cytometry (FACScan flow cytometer; BD Biosciences, San Jose, CA). Cells were stained with FITC-conjugated CD83, PE-conjugated CD86 and PerCP-conjugated HLA-DR mAb’s (BD Biosciences). Release criteria were >70% viable by trypan blue exclusion, and >30% of the large cell gate being CD86^+^ and HLA-DR^+^. DC were pulsed (co-cultured) with either tumor lysate overnight or the HLA-A0201 restricted peptides gp100_209–217(209M)_, TRP-2_180–188_, Survivin_96–104_, Her-2/neu_369–377_(ClinAlfa/Biosynthesis, Inc.) and KLH (biosyn Corporation) for 90 minutes prior to washing and injection. The final product was tested for sterility by Gram stain, mycoplasma and endotoxin prior to injection.

### Treatment Schema

Newly diagnosed glioblastoma patients underwent surgery and a standard course of external beam radiotherapy with concurrent temozolomide chemotherapy prior to DC vaccination [4]. These patients were given three biweekly DC vaccinations following standard chemo-radiation and prior to adjuvant temozolomide treatment. Recurrent glioblastoma patients had previous radiation therapy and chemotherapy prior to presenting with tumor recurrence, so they underwent surgical resection of their tumors followed by DC immunotherapy after they had recovered from surgery and were tapered off peri-operative steroids.

### Vaccine Administration

On the day of each DC vaccination, a 1 ml vaccine dose was drawn into a sterile tuberculin syringe and administered as an intradermal (i.d.) injection (using a 25-gauge needle) in the arm region below the axilla, with the side of administration rotated for each vaccination. Subjects were monitored for two hours post-immunization in the UCLA General Clinical Research Center (GCRC). Eligible patients initially received three (3) intradermal injections at biweekly intervals. All patients had a baseline brain MRI scan within one month prior to starting the immunotherapy and every two months thereafter or when clinically indicated.

### Collection of PBMC for Immune Monitoring

Peripheral blood was drawn from patients at various time points both pre and post DC vaccination. Once drawn, the peripheral blood was diluted in a 1∶1 dilution of HBSS Media and PBMCs were harvested through subsequent extraction with Ficoll. After a series of three washes in HBSS media, the cells were placed in a freezing media of 10% DMSO and 90% serum and stored in liquid nitrogen.

### Antibody Staining of PBMC

Normal donor PBMC and patient PBMC were thawed at 37°C for five minutes then immediately transferred to 10 ml RPMI media and subsequently centrifuged at 4°C at 1070 RPM for five minutes to remove DMSO from the freezing solution. The cell pellet was resuspended with 5 ml RPMI and counted using a hemocytometer and a light microscope. After counting, the samples were centrifuged using the same settings and resuspended at five million cells/ml PBS.

A normal PBMC negative control (one well), single color compensation controls (eight wells), and experimental samples (one well/sample) were then plated at one million cells per well (200 µl) in a 96-well round bottom plate. In our experiment, two sets of the experimental samples were plated, as two different antibody cocktails (an eight-color combination and a seven-color combination) were necessary to investigate all of the surface markers of interest due to the spectral limitations of the flow cytometer. The plate was then centrifuged (same settings), decanted and resuspended in prepared antibody cocktails and stained in the dark on ice for 30 min. Antibody cocktails were prepared according to manufacturer specifications, usually 5 µl antibody/50 µl FACS buffer for single color compensation controls. The negative control was resuspended in 50 µl staining buffer. The eight-color combination antibody cocktail contained CD3 Texas Red (Invitrogen, Cat. MHCD0317), CD4 Alexa Fluor 700 (BD Biosystems, Cat. 557922), CD8 Pacific Blue (BD Biosystems, Cat. 558207), CD16 FITC (BD Biosystems, Cat. 555406), CD19 PE (BD Biosystems, Cat. 555413), CD25 APC Cy7 (BD Biosystems, Cat. 557753), CD127 AF647 (BD Biosystems, Cat. 558598), and CD69 PE-CY5 (BD Biosystems, Cat. 555532) antibodies in the same amount used in the single color control in 50 µl staining buffer/patient. The seven-color combination antibody cocktail contained CD3 Texas Red (Invitrogen, Cat. MHCD0317), CD4 Alexa Fluor 700 (BD Biosystems, Cat. 557922), CD8 Pacific Blue (BD Biosystems, Cat. 558207), CD16 FITC (BD Biosystems, Cat. 555406), CTLA-4 PE (BD Biosystems, Cat. 555853), PD-1 AF647 (eBioscience, Cat. 51-9969-73), and CD69 PE-CY5 (BD Biosystems, Cat. 555532) antibodies in the same amount used in the single color control in 50 µl staining buffer/patient. The plate was then centrifuged (same settings), decanted and resuspended with IC-Fixation buffer and stored in the dark on ice for ten minutes. The plate was then centrifuged (same settings), decanted and resuspended in 200 µl staining buffer. Samples were then transferred to labeled flow tubes with an additional 200 µl staining buffer, capped and stored at 4°C.

### Flow Cytometric Analysis of PBMC

All samples were analyzed using the BD LSR II flow cytometer and BD FACS diva software. Cytometer settings were set such that only the eight colors used were present. The negative control was then acquired and FSC and SSC voltages were adjusted such that the lymphocyte population was visible. The single color compensation controls were then acquired and voltages were adjusted such that the stained cells did not exceed 10^4^. A gate was set on stained controls in comparison to the negative control and then each sample was recorded and all compensation controls were applied. Samples were then recorded at 500,000 events per sample. Data acquired from the flow cytometer was analyzed using FlowJo software. The lymphocyte population was gated and then the lymphocyte subsets were gated: CD3^+^CD4^+^ Helper T cells, CD3^+^CD8^+^ cytotoxic T cells, CD3^-^CD16^+^ classical Natural Killer (NK) cells, CD3^+^CD16^+^ NKT cells, CD3^-^CD19^+^ B cells, CD3^+^CD25^+^CD127^low^ Treg cells. The activation status and expression of negative co-stimulatory molecules on these PBMC were also evaluated by measuring the frequency of CD25, CD69, CTLA-4 and PD-1.

### Statistical Analysis

The raw data was aggregated and tabulated for analysis and study using GraphPad software v5.03 (GraphPad Software, La Jolla, CA). Descriptive statistics such as mean and standard deviation were used to summarize continuous variables, while count and percentage were used for categorical variables. Bivariate comparisons of continuous variables and categorical variables were performed using unpaired t-tests or Fisher’s exact tests, respectively. The Kaplan Meier method and log-rank test were used to summarize and compare the overall survival and time to progression between trials. A univariate Cox proportional hazards regression model was used to correlate the individual immune monitoring variables with overall survival. A recursive partitioning survival tree was built to obtain the cut-off values utilized to dichotomize each immune monitoring variable, which could differentiate the overall survival between the variables. For all statistical investigations, tests for significance were two tailed, with a statistically significant p-value threshold of 0.05. Statistical analyses were performed using SAS 9.2 (SAS institute, Cary, NC).

## Results

### Patient Characteristics

**Figure 1:**
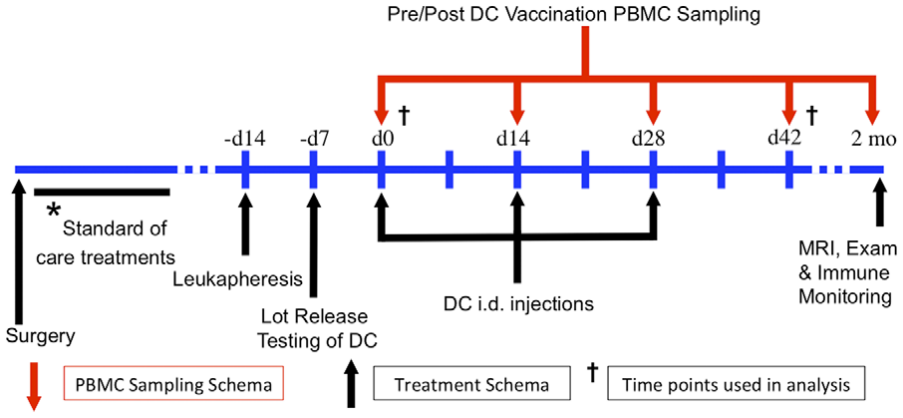
Treatment and PBMC sampling schema of newly diagnosed and recurrent GBM patients. *Surgery, external beam radiotherapy (XRT) and concurrent temozolomide chemotherapy was given per standard of care prior to DC vaccination in newly diagnosed glioblastoma patients. Recurrent patients were treated with surgical resection and then enrolled immediately to the DC trial. Such patients did not receive concurrent XRT and temozolomide. Leukapheresis was performed two weeks prior to vaccination followed by preparation of the DC vaccine one week prior to vaccination. Three doses were administered intradermally (i.d.) at biweekly intervals. A follow up MRI was then performed two months after the start of immunotherapy or when clinically indicated. PBMC sampling was performed by peripheral blood draw prior to each vaccination and again at day 42.

Twenty-four patients with histologically diagnosed glioblastoma (WHO Grade IV) were enrolled and treated in two Phase 1 clinical trials at UCLA. There were five women and nineteen men, with an age range from 27 to 71 years of age (mean age of 48 years). Nineteen patients underwent treatment with autologous tumor lysate pulsed DC vaccination (ATL DC) while five patients underwent GAA peptide pulsed DC immunotherapy (GAA DC). There was no significant age difference in the ATL DC versus the GAA DC cohorts (48 vs. 43 years, p = 0.52). **Figure 1** outlines the treatment schema used in these two clinical trials.

### Overall Survival and Time to Tumor Progression

The median overall survival (OS) in the ATL DC vaccine group was 33.8 months, while that of the GAA DC group was 14.5 months. Several of the ATL DC patients were long-term survivors whereas all of the patients undergoing GAA DC therapy died during the follow up period. The median time to tumor progression (TTP) in the ATL DC vaccine therapy cohort was 13.9 months, while that of the GAA DC vaccine cohort was 9.6 months. The median OS and TTP for both groups together was 23.0 and 13.1 months, respectively.

### Lymphocyte Activation Markers are not Associated with Survival after DC Vaccination in Glioblastoma Patients

**Figure 2:**
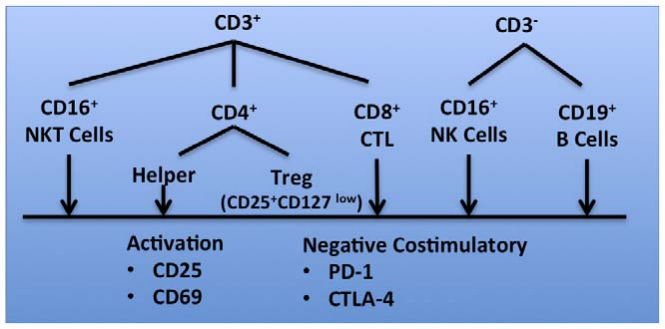
FACS-based immune monitoring strategy in DC vaccine patients. PBMC were isolated by ficoll separation prior to, and after three bi-weekly DC vaccinations, frozen, and subsequently thawed for staining simultaneously. Using a multi-color mAb cocktail, six distinct lymphocyte populations were identified. In each population, the expression of activation markers (CD69, CD25) and negative co-stimulatory factors (PD-1, CTLA-4) were also evaluated.

Using a multi-color panel of fluorescently conjugated antibodies, we were able to differentiate six specific cell populations (CD3^+^CD4^+^ helper T cells, CD3^+^CD8^+^ cytotoxic T cells, CD3^-^CD16^+^ NK cells, CD3^+^CD16^+^ NKT cells, CD3^-^CD19^+^ B cells, CD3^+^CD4^+^CD25^+^CD127^low^ regulatory T cells) within the peripheral blood of patients in both the ATL and GAA peptide DC trials (**Figure 2**). In each cell subset, we evaluated the expression of activation (CD25, CD69) and negative costimulatory markers (PD-1, CTLA-4). To account for the heterogeneity in patient PBL populations before, and after DC vaccination, comparisons were made from pre to post DC vaccination for each patient, in order to create fold changes. We then examined these fold changes in each lymphocyte subset and looked for statistically significant relationships.

No significant changes were observed in any lymphocyte subset frequency before and after DC vaccination (**Table 1**). In a recent DC vaccination study, Ardon, et al., could not find any positive correlation between immune reactivity and clinical outcome in patients vaccinated with autologous tumor lysate-pulsed DC vaccination [23]. Similarly, we also did not find any statistically significant relationship between the activation status of any lymphocyte subsets and survival (data not shown). These results suggest that a complex interplay may be involved in the immunoregulatory aspects of the overall anti-tumor immune response.

**Table 1:** Lymphocyte Subset Changes (Pre-Post) DC Vaccination.

Lymphocyte Subset*	Pre-Tx (Avg %)	Post-Tx (Avg %)
CD3^+^CD4^+^ Helper T cells	38.8	37.6
CD3^+^CD8^+^ CTL	25.9	24.4
CD3^+^CD16^+^ NK T cells	5.4	4.7
CD3^−^CD16^+^ NK cells	11.5	14.1
CD3^−^CD19^+^	9.5	9.9
CD3^+^CD4^+^CD25^+^CD127^low^ Treg	19.1	18.2

* Percent of cells stained from ficoll-isolated PBMC at each time point.

### Changes in Regulatory T cell Populations are Associated with Extended Survival in Glioblastoma Patients after DC Vaccination

**Figure 3:**
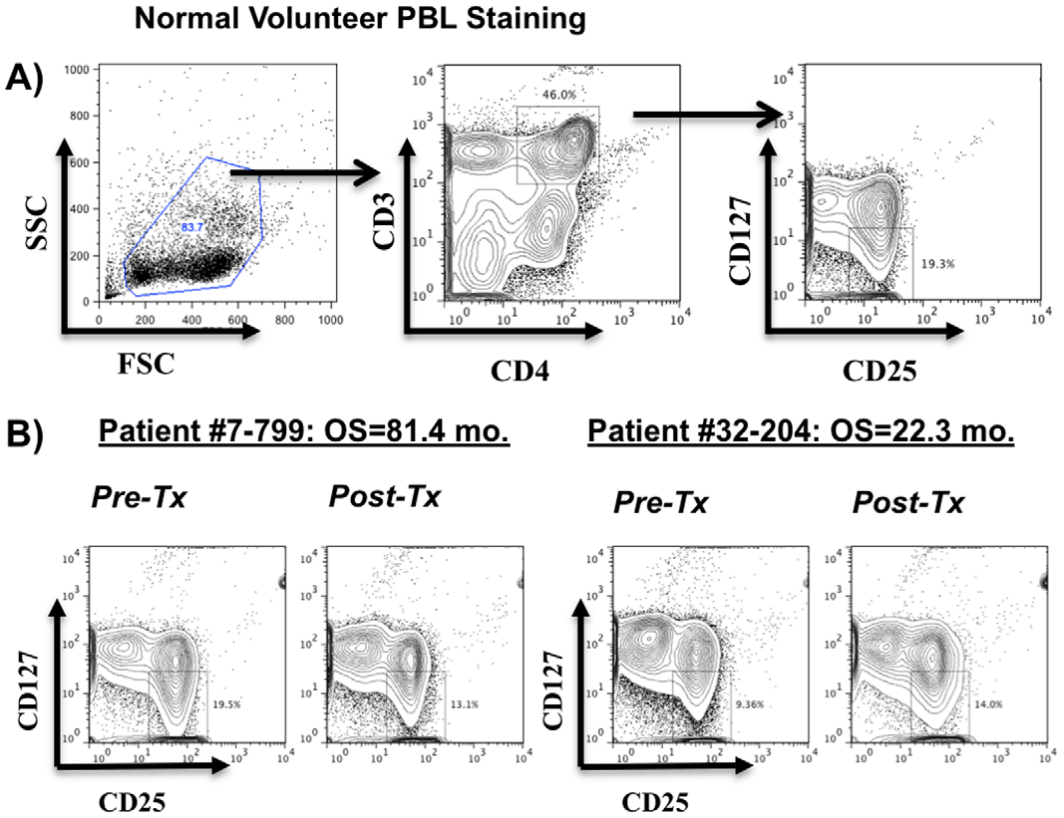
Decreased frequencies of Treg cells after DC vaccination are associated with extended survival. PBMC from pre and post-DC vaccination time points were stained with an antibody cocktail that identifies Treg cell populations (CD3^+^CD4^+^CD25^+^CD127^low^). The ratio of post vaccination/pre-vaccination Treg cell frequencies from each patient was calculated and linked with the overall survival of each patient. (A) Flow cytometric analysis of Treg cell populations from a normal volunteer. (B) Representative FACS plots of Treg cell frequencies from two glioblastoma patients (7–799, 32–204) before and after DC vaccination.

Pre-clinical models of glioma have suggested that regulatory T (Treg) cell populations can impact anti-tumor immune responsiveness [25], [28], [29], [31], [36], [37] and pilot clinical studies suggest that depletion of Treg cells in glioblastoma patients may be associated with enhanced vaccine efficacy [38]. However, no published literature has examined whether Treg cell populations are altered in glioblastoma patients after DC vaccination, and whether any differences have clinical ramifications. We used a cell surface staining protocol to identify Treg cell populations in human PBL [39], [40] so that we could track this cell population before and after DC vaccination in malignant glioma patients (**Figure 3**). We then evaluated whether the fold change of Treg cell frequency was associated with survival in these patients. Using a univariate Cox proportional hazards model, we discovered a highly significant relationship between Treg cell frequency changes and survival (hazard ratio = 3.623; 95% C.I. (1.196, 10.976); **Table 2**). Based on this statistical assessment, every single unit increase in the Treg cell ratio is associated with an increased risk of death by 2.623 times (3.623-1). This increase is statistically significant (p = 0.0228). These findings suggest that extended survival is observed in patients whose Treg cell frequency decreases after DC vaccination.

**Table 2:** Univariate Cox proportional hazards model for overall survival with each immune monitoring ratio.

Variable	Cut-off point	P-value for log-rank test (OS)
Treg cells (dichotomized)	0.8865	0.0074 (See Fig. 5a)
CD4^+^ T cells w/CTLA-4 (dichotomized)	1.047	0.0034 (See Fig. 5b)
CD8^+^ T cells w/CTLA-4 (dichotomized)	0.8065	0.0105 (See Fig. 5c)
Activated CD8^+^ T cells (dichotomized)	0.703	0.0926

### Dynamic Changes in the Expression of CTLA-4 are Associated with Survival in Glioblastoma Patients Treated with DC Vaccination

**Figure 4:**
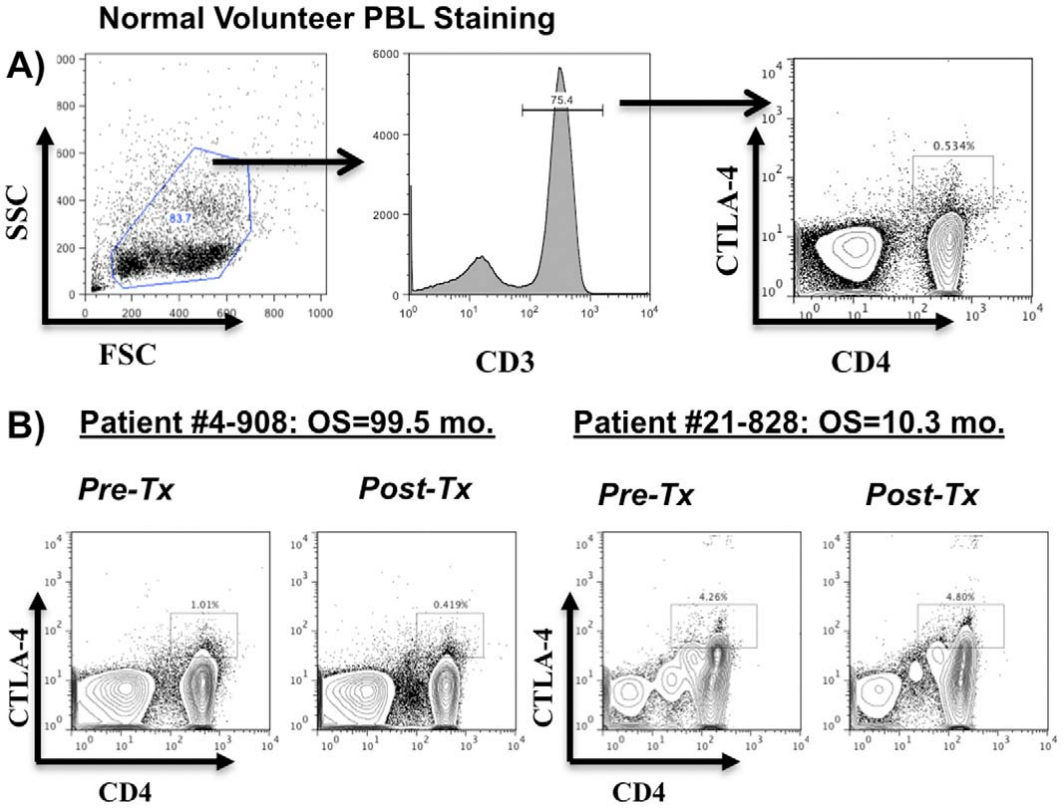
Decreased expression of CTLA-4 on CD3^+^CD4^+^ and CD3^+^CD8^+^ T cells after DC vaccination is associated with extended survival. PBMC from pre and post-DC vaccination time points were stained with an antibody cocktail that identifies CD4 and CD8 T cell populations (CD3^+^CD4^+^or CD3^+^CD8^+^) and evaluated for the expression of CTLA-4. The ratio of post vaccination/pre-vaccination expression of CTLA-4 from each patient was calculated and linked with the overall survival of each patient. (A) Flow cytometric analysis of CD3^+^CD4^+^ T cell expression of CTLA-4 from a normal volunteer. (B) Representative FACS plots of CTLA-4 expression from CD3^+^CD4^+^ T cells in two glioblastoma patients (4–908, 21–828) before and after DC vaccination.

The inhibitory costimulatory molecules, CTLA-4 and PD-1, are known to impact anti-tumor immune responsiveness in murine tumor models (reviewed in [33]), including gliomas [41]. In fact, the use of a blocking antibody to CTLA-4 (Ipilumimab) in metastatic melanoma patients demonstrated clinical efficacy [7] and was recently given approval by the FDA [9]. To test whether the expression of negative co-stimulatory molecules was altered in glioblastoma patients receiving DC vaccination, we stained pre and post vaccination PBL. The expression of PD-1 on any PBL subset was not significantly altered after DC vaccination and did not correlate with survival in these patients (data not shown). However, a significant association with survival was discovered for the expression of CTLA-4 by CD3^+^CD4^+^ T cells (Hazard Ratio for death = 2.84; p = 0.0191) and CD3^+^CD8^+^ T cells (Hazard Ratio for death = 2.174; p = 0.0460) in these patients (**Figure 4**, **Table 2**). These findings suggest that extended survival is observed in patients whose expression of CTLA-4 decreases in helper and cytotoxic T cells after DC vaccination.

### Estimated Post/pre Treatment Ratios for Treg Cell Frequencies and CTLA-4 Expression Significantly Dichotomize the Survival of Glioblastoma Patients Receiving DC Vaccination

**Figure 5:**
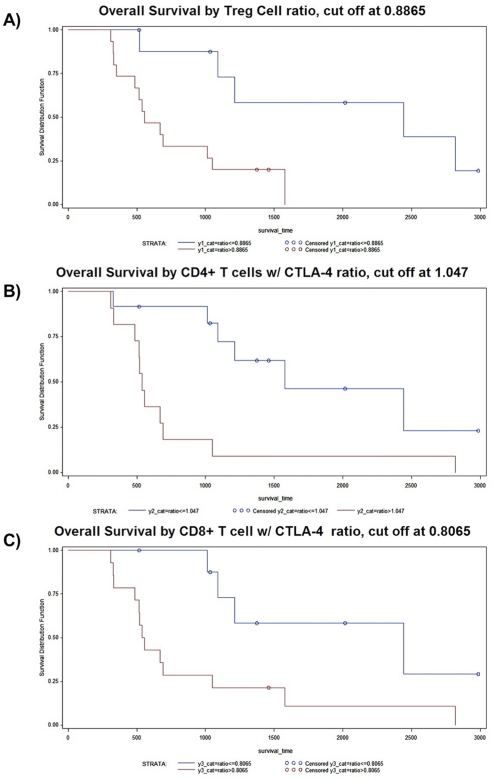
Estimated post/pre treatment ratios for Treg cell frequencies and CTLA-4 expression significantly dichotomize the survival of malignant glioma patients receiving DC vaccination. A recursive partitioning survival tree algorithm was utilized to dichotomize the immune monitoring variables. The overall survival was then compared between patients above and below the cut-off point. (A) Kaplan-Meier survival curve for the overall survival of patients stratified above and below a cut-off value of 0.8865 for Treg populations. **p = 0.0074 by log-rank test. (B) Kaplan-Meier survival curve for the overall survival of patients stratified above and below a cut-off value of 1.047 for the expression of CTLA-4 on CD3^+^CD4^+^ T cells. **p = 0.0034 by log-rank test. (C) Kaplan-Meier survival curve for the overall survival of patients stratified above and below a cut-off value of 0.8065 for the expression of CTLA-4 on CD3^+^CD8^+^ T cells. *p = 0.0105 by log-rank test.

To develop a predictive immune monitoring tool for patients treated with DC vaccination, we utilized a recursive partitioning survival tree to dichotomize the immune monitoring ratio variables. Then, we compared the survival of patients above and below the calculated cut-off. As shown in **Table 3** and **Figure 5**, significant differences in survival were found when DC vaccine patients were stratified by these estimated cut-off values for Treg cell fractions and CTLA-4. These data suggest that DC-vaccinated glioblastoma patients, whose Treg cell frequency or ratio of CTLA-4 expression (post-Tx:Pre-Tx) is below the cut-off value, will have extended survival.

**Table 3:** Dichotomization of overall survival with each immune monitoring ratio.

Variable	Estimated HR	P-value	95% CI
Treg cells	3.623	0.0228	(1.196, 10.976)
CD4^+^ T cells w/CTLA-4	2.840	0.0191	(1.186, 6.798)
CD8^+^ T cells w/CTLA-4	2.690	0.0273	(1.117, 6.474)
Actiated CD8^+^ T cells	1.027	0.9316	(0.561, 1.878)

## Discussion

In the present study, we analyzed the relationship between several immune monitoring variables and the survival of two cohorts of glioblastoma patients treated with dendritic cell vaccination. This study is one of the largest studies reported to date comparing the outcomes of different DC vaccine patient cohorts from a uniform population. Our results strongly suggest that the balance of regulatory T cell frequencies and expression of negative co-stimulatory molecules on peripheral blood T cells can influence relevant anti-tumor immune responses in this patient population. In addition, the development of an estimated post-treatment/pre-treatment cut-off point ratio for each immune monitoring variable may provide a segue to the design of prospectively designed trials where we can test its clinical relevance. As such, these tools can be incorporated and used to evaluate and compare different monitoring strategies.

Our results also suggest that the dendritic cell vaccine platform could be augmented by other biological therapies that influence Treg cell populations and negative costimulatory molecules. Increased post vaccination frequencies of Treg cell populations were associated with shorter survival in our glioblastoma patients who received the DC vaccination (**Table 2**). These findings are consistent with the current understanding that Treg cells play a significant role in down regulating the anti-tumor immune response. Therefore, it follows that patients with lower levels of Treg cells post vaccination should have stronger immune responses and consequently longer survival. In support of this, Mitchell, et al, recently demonstrated that immune responses were dramatically enhanced after dendritic cell vaccination in glioblastoma patients that received IL-2Rα mAb blockade (daclizumab, Roche Pharmaceuticals) and temozolomide chemotherapy [38]. Such data is consistent with recent reports that natural Treg cells predominate inside gliomas, suggesting that the peripheral depletion of Treg cells may be important for vaccine-elicited anti-tumor immune responsiveness [32]. Similarly, the blockade of CTLA-4 or PD-1 may enhance vaccine-elicited immune responses and anti-tumor activity [42], [43], [44]. Such recent findings lend further credence to the idea that the relative balance between inhibitory immune signals and activation may dictate the overall anti-tumor immune response [24], [45], [46].

Our findings also have clinical applications to other immune therapeutic approaches for glioblastoma. Given the increasingly important role that immunoregulatory factors may play for effective anti-tumor immunity, the dynamic monitoring of Treg cell populations and/or negative co-stimulatory molecule expression on PBL populations before and after therapy may allow groups to monitor treatment efficacy and predict survival. Such an immune monitoring strategy can be generally applicable to various immunotherapeutic strategies [30], as it is non-antigen specific and may give important information regarding the balance of inhibitory and activation signals critical for the anti-tumor immune response.

In conclusion, a statistically significant relationship was found between the fraction of Treg cells or dynamic expression of CTLA-4 in peripheral blood T cells with survival in glioblastoma patients treated with dendritic cell vaccination. These data suggest that decreased ratios of Treg cells or CTLA-4 after DC vaccination may be associated with good prognosis in this patient population. In contrast, there was no significant relationship found between lymphocyte activation markers and survival, suggesting that inhibitory immune checkpoints may dominantly regulate anti-tumor immune responses on peripheral blood lymphocytes after DC vaccination. In future studies, blood drawn from patients before and after treatment and can be used as a means of predicting their response to various treatment modalities. Adjuvant therapies targeting regulatory T cells may potentially be used in conjunction with DC vaccine immunotherapy to augment the immune response to such therapies.
