# Correction: Monitoring of Regulatory T Cell Frequencies and Expression of CTLA-4 on T Cells, before and after DC Vaccination, Can Predict Survival in GBM Patients

**DOI:** 10.1371/annotation/641c3e73-ee2c-4873-8896-1b3a2edc557b

**Published:** 2012-04-13

**Authors:** Brendan Fong, Richard Jin, Xiaoyan Wang, Michael Safaee, Dominique N. Lisiero, Isaac Yang, Gang Li, Linda M. Liau, Robert M. Prins

There was an error in the placement of Tables 2 and 3. The Tables were switched. 

